# Graphene-based terahertz reconfigurable printed ridge gap waveguide structure

**DOI:** 10.1038/s41598-022-23861-y

**Published:** 2022-12-06

**Authors:** Mohamed Mamdouh M. Ali, Shoukry I. Shams, Mahmoud Elsaadany, Ghyslain Gagnon, Ke Wu

**Affiliations:** 1 Faculty of Engineering, Department of Electrical Engineering, Assiut University, Assiut, Egypt; 2 Department of Electrical and Computer Engineering, Concordia University, Montreal, QC Canada; 3 Electrical Engineering Department, Ecole de Technologie Superieure, Montreal, QC Canada; 4 Department of Electrical Engineering, PolyGrames Research Center, Ecole Polytechnique de Montréal, Montreal, Canada

**Keywords:** Engineering, Electrical and electronic engineering

## Abstract

Graphene-based microwave devices have enabled reconfigurability, thus paving the way to the realization of flexible wireless terahertz systems with featured performances. Despite great progress in the development of graphene-based terahertz devices in the literature, high insertion loss and wide tunable range are still significant challenges at such high frequencies. In this work, we introduce the use of graphene to implement a reconfigurable printed ridge gap waveguide (RPRGW) structure over the terahertz frequency range for the first time. This guiding structure is suitable for both millimeter and terahertz wave applications due to its supporting quasi-TEM mode, which exhibits low dispersion compared to other traditional guiding structures. The presented solution is featured with low loss as the signal propagates in a lossless air gap, which is separated from the lossy graphene elements responsible for the reconfigurable behavior. In addition, this guiding structure is deployed to implement a tunable RPPGW power divider as an application example for the proposed structure.

## Introduction

Reconfigurable systems are gaining considerable attention as they are expected to play an important role in the development of terahertz wireless technologies. Reconfigurability can result in flexible, cost-effective, and smart transceivers overcoming the design challenges at such high frequencies and achieving the technological future demands^1^. Reconfigurable systems can be constructed through deploying sub-systems having the ability to reversibly change their functionality under some controlling process. One of the major revolutions of reconfigurability in electronics is the field-programmable gate array (FPGA), which has a powerful impact on digital design methodologies alongside wireless systems^2,3^. In a related context, few trials are initiated to develop reconfigurable microwave components providing an additional level of functionality for wireless systems. These components can be implemented using a traditional guiding structure such as a microstrip line, rectangular waveguide, and substrate integrated waveguide^4–6^. However, these guiding structures have high material losses and fabrication limitations over higher frequency bands. Therefore, the research community exerts more effort to develop innovative guiding structures such as ridge gap waveguide (RGW) to overcome the high frequency limitations and satisfy the technical requirements for future wireless technology^7–9^. RGW is considered as a promising guiding structure for millimeter and terahertz wave applications due to the recently reported advantages such as supporting the propagation of quasi-TEM (Q-TEM) mode with lower loss compared with other guiding structures^10–14^.

Reconfigurability of microwave components can be achieved through various mechanisms such as switches, variable reactive loading, and mechanical and material variation. The most common technique used is deploying a switch such as MEMS, PIN diode, and field-effect transistor (FET), which can alter the performance of components in a discrete manner^15–19^. One of the promising techniques to realize these switches are using graphene. Graphene has incited great interest due to its attractive features that lead to potential usage in different applications^20–22^. Graphene’s transport characteristics and conductivity can be tuned by either electrostatic or magnetostatic gating, or via chemical doping, thereby leading to the possibility of developing various electronic devices^23–25^. Based on the possibility of controlling the graphene conductivity (impedance) by field effects, several studies have been conducted showing a possibility to reconfigure the performances of microwave devices and antennas^26–33^. However, few studies have addressed the effect of tuning the graphene on the frequency response of guiding structures such as coplanar waveguide, parallel plate waveguide, rectangular waveguide, microstrip line, and substrate integrated waveguide^30,34–40^.

To our best knowledge, the use of graphene to implement a reconfigurable printed ridge gap waveguide (RPRGW) at terahertz is proposed for the first time in this paper. The PRGW structure offers the separation between the graphene and the signal path, which imply by nature the low loss characteristics compared with SIW graphene-based structure as the graphene exists in the signal path^6,36^. A reconfigurable Electromagnetic Band Gap (EBG) unit cell is investigated to construct the PRGW guiding structure. Simulated results show that inserting graphene in the EBG unit cells influences the higher frequency of the band gap. This is achieved by altering the chemical potentials of the loaded graphene. Thus, the operating bandwidth of the PRGW line can be varied corresponding to changing the chemical potentials of graphene. As an application of the proposed RPRGW line, a wideband reconfigurable PRGW power divider is simulated. The results verify that the proposed RPRGW can provide an effective guiding structure for reconfigurable terahertz devices.

## Electronic model of graphene

The aforementioned applications and challenges in the terahertz motivate the scientific community to develop novel solutions that can overcome significant losses and provide reconfigurable features. We introduce for the first time the deployment of graphene in the printed ridge gap waveguide technology to enable the reconfigurable behavior of the entire structure. We propose the placement of the graphene in the cell structure away from the signal path to avoid the loss of contribution of the graphene, which will be discussed in detail in the following section. Before going through the proposed structure, we will give a brief discussion about the graphene material characteristics and modes of operation.

Many articles have discussed the electronic model of graphene, which can be modeled as an infinitesimally thin surface characterized by a surface conductivity $\sigma (\omega ,\mu _c,\Gamma ,T)$, where $\omega$ is radian frequency, $\mu _c$ is chemical potential, $\tau$ is electron relaxation time, $\Gamma =\frac{1}{\tau }$ is electron scattering rate, and *T* is temperature in kelvin^23–31,34–37^. The chemical potential is determined by the carrier density, where varying the carrier density will affect the mobility of the electron-hole, thus changing the material conductivity. The carrier density can be controlled by the application of a gate voltage, wherein in the ungated case both the carrier density and chemical potential can be considered zero^24^. The conductivity of graphene $\sigma$ has been studied in several recent works and can be expressed as the summation of two complex quantities, namely, intra-band and inter-band conductivity. These two quantities can be expressed as:

1 $$\begin{aligned} \sigma _{intra}= & {} \frac{-2j e^2 k_B T}{\pi h'^2 (\omega -j \Gamma )} ln\left( 2cosh\left( \frac{\mu _c e}{2k_B T}\right) \right) \end{aligned}$$

2 $$\sigma _{inter}= \frac{-j e^2}{4 \pi h'} ln\left( \frac{2 \vert \mu _c\vert -(\omega -2j\Gamma )h'}{2 \vert \mu _c\vert +(\omega -2j\Gamma )h'}\right)$$

where *e* = 1.60217662 × 10$^{-19}$ is the electron charge, $k_B$ = 1.38064852 $\times$ 10$^{-23}$ is the Boltzmann constant, and $h'$ = $\frac{h}{2 \pi }$ = 1.054 $\times$ 10$^{-34}$ is the reduced Plank constant. Usually, at microwave and millimeter frequencies, the effect of inter-band conductivity is negligible, whereas the intra-band conductivity is solely considered.

**Figure 1:**
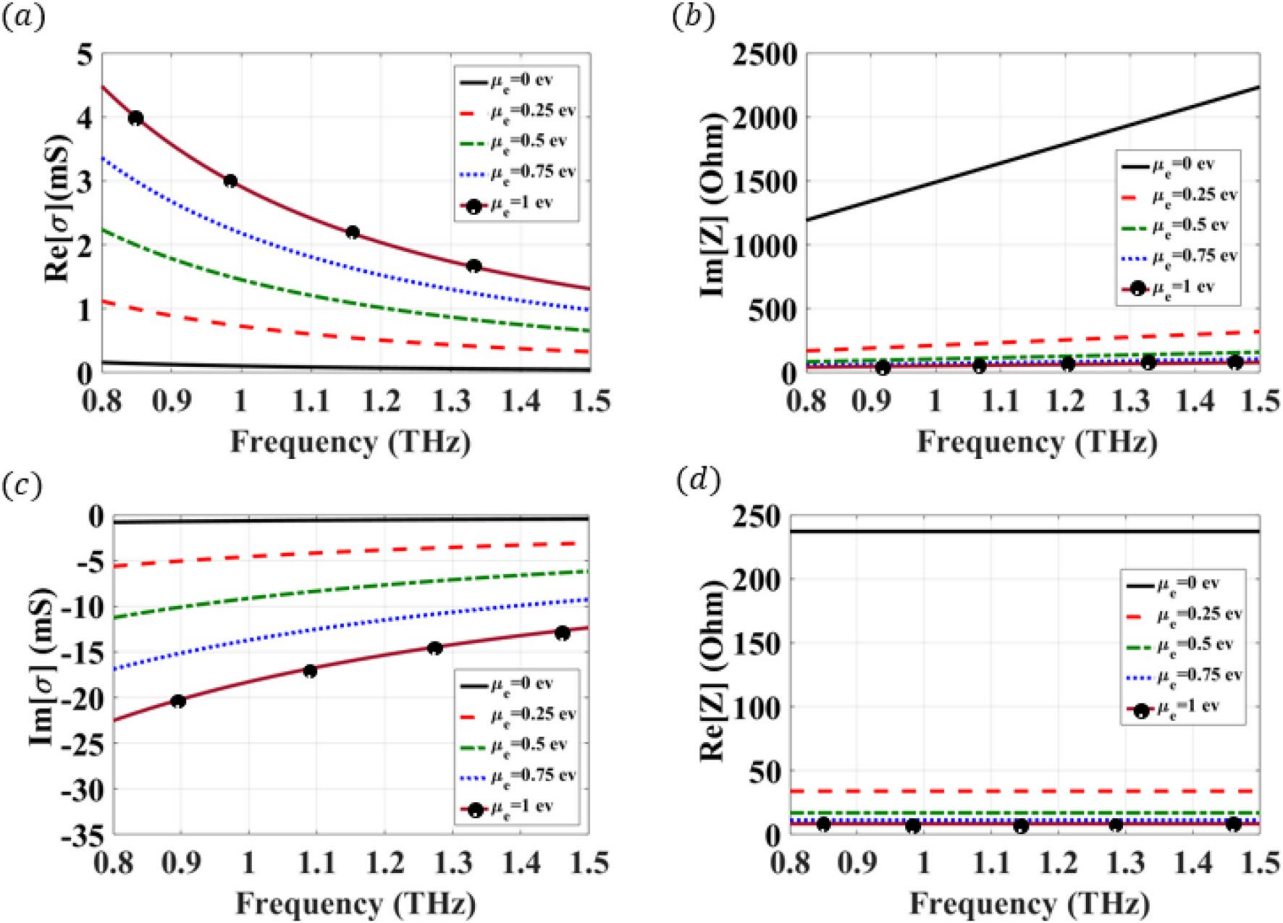
Graphene conductivity and surface impedance evaluated for several chemical potentials from $\mu _c=0$ ev to $\mu _c=1$ ev. (**a**) conductivity real part. (**b**) conductivity imaginary part. (**c**) surface impedance real part. (**d**) surface impedance imaginary part.

The effect of chemical potential variation of the graphene’s real and imaginary conductivity is studied and plotted in Fig. 1a,b. An electron relaxation time of 1 ps, a temperature of 300 K, and a chemical potential $\mu _c$ variation from 0 to 1 ev are assumed, where these values are used for THz frequencies. The conductivity of graphene is directly proportional to this relaxation time, where high conductivity can be obtained using relatively long relaxation times. Therefore, this range of relaxation time is associated with a reasonable conductivity value. This phenomenon has been visited in multiple articles, where the same range of relaxation time has been assumed in the literature^26,41^. It can be noticed that the imaginary part of the conductivity is negative, meaning that the graphene can be modeled by a surface impedance $Z=\frac{1}{\sigma } =R+jX$, where the graphene impedance can be evaluated as^24,42^:

3 $$\begin{aligned} R (\mu _c)= & {} \frac{\pi \Gamma h'^2}{2 e^2 k_B T ln\left( 2cosh\left( \frac{\mu _c e}{2k_B T}\right) \right) } \end{aligned}$$

4 $$\begin{aligned} X (\mu _c)= & {} \frac{\pi \omega h'^2}{2 e^2 k_B T ln\left( 2cosh\left( \frac{\mu _c e}{2k_B T}\right) \right) } \end{aligned}$$

The real and imaginary part of the graphene impedance is plotted in Fig. 1c,d, which demonstrates that the graphene impedance is increasing by decreasing the chemical potential $\mu _c$, where the maximum surface resistance of a graphene sheet is achieved at $\mu _c=0$ ev. Therefore, the graphene impedance can be considered as an open and short circuit when $\mu _c=0$ ev and $\mu _c=1$ ev, respectively. As a result, by tuning the chemical potential of graphene, the surface impedance can be altered so to reconfigure the performance of the guiding structure.

## Re-configurable terahertz PRGW structures and applications

Ridge gap waveguide is considered among the emerging guiding structures, introduced for the first time in 2009^8,9^. The signal propagates in the form of a Q-TEM mode between the ridge and the top ground, while the cells prevent any leakage within the operating bandwidth^43^. The cell stop band represents the operating bandwidth of the entire structure. This structure has been investigated intensively in the past decade, where various components have been proposed based on this promising technology^11,44,45^. This technology provides a wide band operation with minimal dispersion, which is suitable for high-frequency applications. In addition, a printed version of the same structure has been proposed to provide the same advantages in a low-cost guiding mechanism. Different unit cell structures have been examined in the literature for (RGW) Ridge Gap waveguides and PRGW—Printed Ridge Gap waveguides^12–14^. In this work, we aim to utilize a novel unit cell structure, where some metallic parts can be replaced by graphene. Afterward, applying a controlling voltage will vary the conductivity of cell parts replaced by graphene. As a result, the effective cell shape will depend on the applied controlling voltage. Hence, the operating bandwidth of the entire structure can be adjusted through the controlling voltage.

### Cell design and analysis

**Figure 2:**
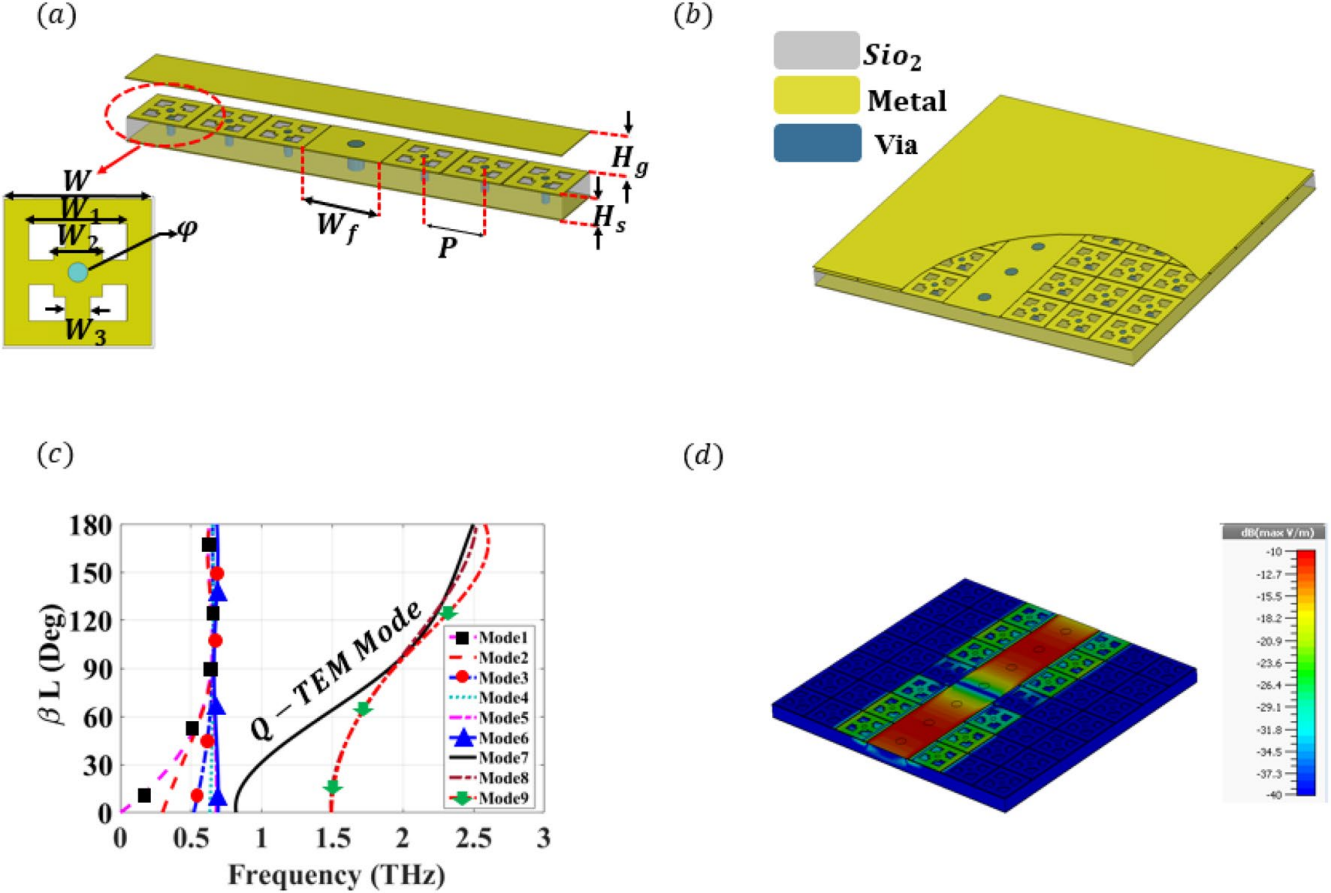
3-D view of the proposed terahertz PRGW transmission line and simulated results of the proposed terahertz PRGW transmission line. (**a**) The 3D view of a PRGW section used to construct the PRGW line. Physical parameters are sets as ridge width $W_f$ = 40 μm, period *P* = 31 μm, substrate height $H_s$ = 10 μm, and air gap height $H_g$ = 2 μm. Unit cell parameters and dimensions are chosen as *W* = 30 μm, $W_1$ = 20 μm, $W_2$ = 10 μm, $W_3$ = 5 μm, and via radius $\phi$ = 4 μm. (**b**) 3D geometry of PRGW line. This line is consisting of a metal strip connected to the bottom ground through vias, which is surrounded by EBG unit cells covered by a ground plane. (**c**) Dispersion diagram of the PRGW section from the Eigenmode solver of CST. (**d**) Electric field distribution for the propagating mode at 1 THz extracted from the transient solver of CST.

First of all, we propose an innovative cell structure to ensure the operation at the target frequency band, where some metallic parts will be replaced by graphene in a later design stage. The proposed cell construction, row of cells, and a ridge surrounded by three successive cells are illustrated in Fig. 2a,b, respectively. The proposed cell is simulated through the CST Microwave Studio, Eigen Mode Solver, to extract the dispersion diagram of the row of cells in the presence of a ridge. These simulation results are shown in Fig. 2c, where the operating bandwidth can be depicted within the range of 0.8–1.5 THz. The cell dispersion diagram highlights that no propagation mode exists within the frequency band of interest, where the cell can suppress any leakage and ensure wave confinement. On the other hand, the ridge presence creates a Q-TEM mode propagating within the cell stop band. To ensure the field confinement, we simulated a straight line based on the printed ridge gap waveguide technology with CST Microwave Studio Frequency Domain Solver, where the field distribution is shown in Fig. 2d. This figure highlights that the signal is transmitted with full confinement of fields within the ridge region.

### Graphene doped cell: enabling re-configurability

**Figure 3:**
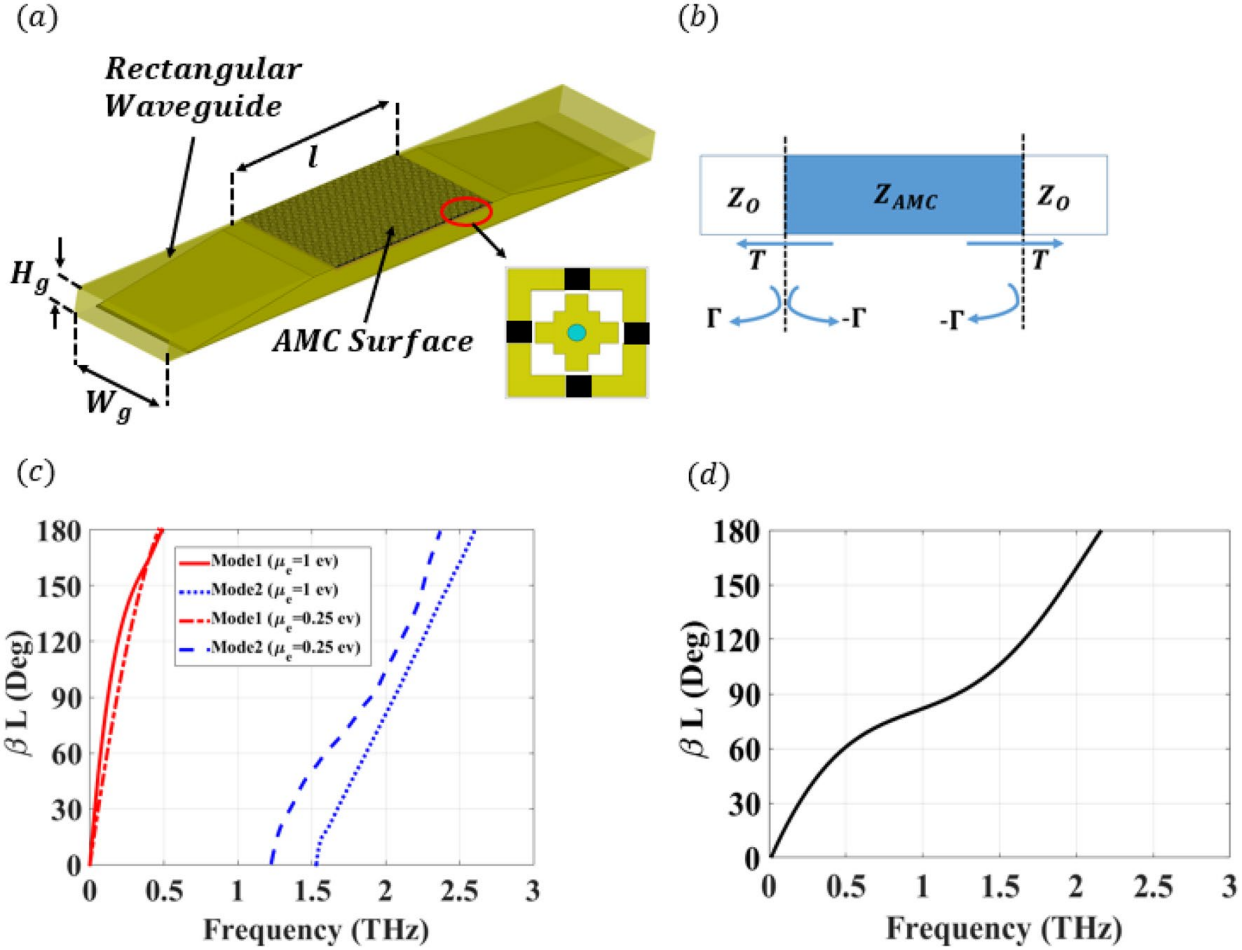
Dispersion diagram extraction and simulated performance. (**a**) 3-D view of the dispersion diagram extraction setup for the graphene-based unit cell. The rectangular waveguide has a cross-section dimensions of $W_g$ = 800 μm and $H_g$ = 200 μm. (**b**) Block diagram of the proposed dispersion diagram extraction setup for the graphene-based unit cell. (**c**) Dispersion diagram of the unit cell for different values of chemical potentials of $\mu _c=1$ ev and $\mu _c=0.25$ ev. (**d**) Dispersion diagram of the unit cell for chemical potentials of $\mu _c=0$ ev.

The proposed cell is modified by adding the graphene material in four rectangular cuts within the cell perimeter to provide tuning elements. It is worth mentioning that the band gap of the conventional unit cell can be determined using the eigenmode solver, which is a numerical technique available in many 3D EM analysis software packages such as CST and HFSS. However, the eigenmode solver packages do not support lossy materials solution, accordingly, they fail to calculate the dispersion diagram of the proposed graphene doped unit cell. Other solvers can be used to solve the lossy materials unit cell dispersion diagram however, a mathematical method based on the scattering parameters is proposed to extract the dispersion relation and study the effect of the graphene material on the band gap of the unit cell^7^. The dispersion relation extraction setup is shown in Fig. 3a, where the periodic graphene-doped rows of cells are placed inside a rectangular waveguide. The waveguide is designed to have a cutoff frequency of 0.2 THz, which is smaller than the lowest operating frequency. Due to the difference in the height between the waveguide and the proposed unit cell, a tapered transition is used to eliminate the discontinuity and provide an accurate evaluation of the reflection and transmission coefficients. This structure can be modeled as a section of unit cells cascaded by two traditional rectangular waveguides from both sides as illustrated in Fig. 3b, where the wave impedance inside of a section of unit cells is assumed to be $Z_{AMC}$, while the waveguide impedance is $Z_o$. The incident wave is routed through the interface between the input port and the section of unit cells, where it is subjected to an infinite number of reflections between both faces. Hence, the expression of magnitudes of the scattering parameter can be given as^7^:

5 $$\begin{aligned} |S_{11}|= & {} \sum |\Gamma _m|\frac{\sqrt{2-2 cos(2\beta l)}}{\sqrt{1+|\Gamma _m|^2-2|\Gamma _m|cos(2\beta l+\theta _{\Gamma _m})}} \end{aligned}$$

6 $$\begin{aligned} |S_{21}|= & {} \sum \frac{{1-|\Gamma _m|}}{\sqrt{1+|\Gamma _m|^2-2|\Gamma _m|cos(2\beta l+\theta _{\Gamma _m})}} \end{aligned}$$

where $\beta l$ is the propagation phase along the Artificial Magnetic Conductor (AMC) surface implemented using the proposed unit cell with length *l*, and *m* is the mode number, while $\Gamma _m$ represents the reflection coefficient at the surface between two hypothetical regions (one has an air-filled dispersion relation and the other has an unknown dispersion relation).

To obtain the dispersion relation, the simulated scattering parameters are obtained using the model shown in Fig. 3a. Afterward, the minima and maxima locations in $|S_{11}|$ and $|S_{21}|$ exist when the phase constant is equal to multiple integers of $\pi$, where the value of $2\beta l$ starts with 0 at the starting propagating frequency. Applying the same process after each bandgap for different values of the chemical potential, the dispersion relation can be obtained as shown in Fig. 3c. It can be depicted from this figure that decreasing the chemical potential voltage from $\mu _c=1$ ev to $\mu _c=0.25$ ev changes the bandgap bandwidth by shifting the cutoff frequency of the higher propagating mode (Mode 2) from 1.5 to 1.2 THz. In addition, at a chemical potential voltage of $\mu _c=0$ ev, the dispersion relation highlights no band gap as shown in Fig. 3d. It can be noticed that there are dramatic band gap changes from $\mu _c=$ 0 to 0.25 eV resulting from the graphene surface impedance behavior that is studied in Fig. 1, where a huge change occurs for the impedance according to the chemical potentials variations. It is worth mentioning that, the proposed method calculates an effective value of the dispersion relation in the case of multi-modes. Accordingly, the proposed method calculates an effective curve for all the modes before the bandgap entitled (Mode1). On the other hand, all the modes after the bandgap are effectively represented by Mode2. In addition, when no bandgap occurs ($\mu _c=$ 0), the entire frequency band has a corresponding value for $\beta l$, where the effective value is calculated over the entire band as shown in Fig. 3d.

**Figure 4:**
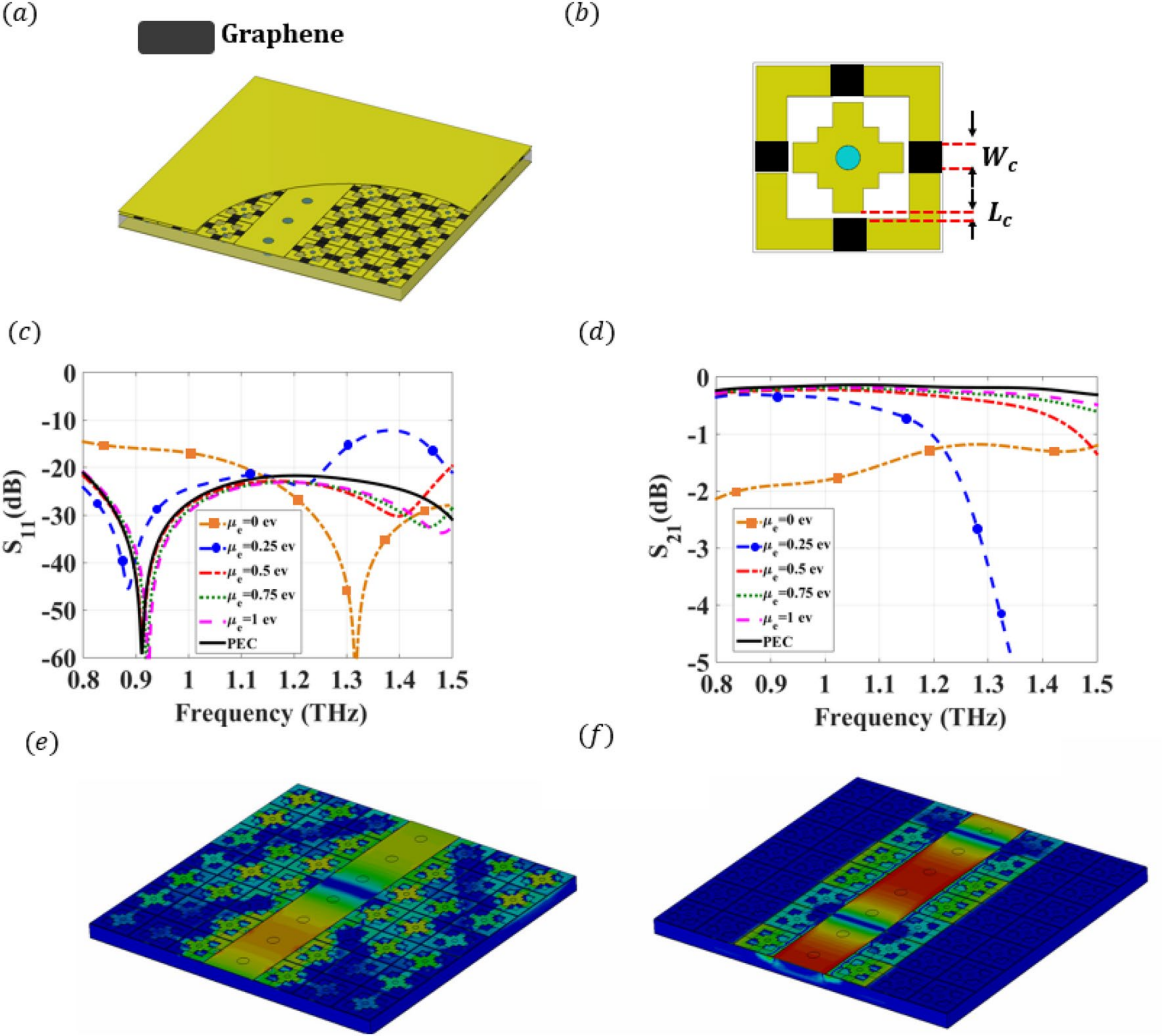
3-D view of the proposed graphene-based terahertz PRGW transmission line and simulated performance using full wave analysis. (**a**) 3-D view of the reconfigurable PRGW line surrounded by a graphene-based unit cell. (**b**) 2-D configuration of the Graphene unit cell. The openings are introduced in the unit cell to allow placing the graphene, where the opening dimensions are set as $W_c$ = 5 μm and $L_c$ = 1 μm. (**c**) Simulated $S_{11}$ of the proposed reconfigurable graphene-based terahertz PRGW transmission line for altering the chemical potentials from $\mu _c=0$ ev to $\mu _c=1$ ev. (**d**) Simulated $S_{21}$ of the proposed reconfigurable graphene-based terahertz PRGW transmission line for altering the chemical potentials from $\mu _c=0$ ev to $\mu _c=1$ ev. (**e**) Electric field distribution for the propagating mode at 1 THz for $\mu _c=0$ ev. (**f**) Electric field distribution for the propagating mode at 1 THz for $\mu _c=1$ ev.

The same outcomes are obtained through the simulated response of the model presented in Fig. 4a, with the cell shown in Fig. 4b. The scattering parameters of this straight line are illustrated in Fig. 4c,d. These figures represent both the return loss and the insertion loss of a straight graphene-doped RGW for different values of the chemical potential. The bandwidth covered at $\mu _c=0$ ev is from 0.8 to 1.5 THz. This band is decreased to be 0.8–1.2 THz at $\mu _c=0.25$. On the other hand, the transmission is lost over the entire band, while zero chemical potential is applied. This study illustrates the controlling voltage impacts on the operation. As another step of verification, the electric field distribution is calculated and plotted in Fig. 4e,f for $\mu _c=0$ ev and $\mu _c=1$ ev , respectively. It can be depicted from these figures that leakage occurs at $\mu _c=0$, while the field is confined within the ridge when the $\mu _c$ reaches 1 ev. In the following part, a dynamic power divider is proposed based on the graphene-doped RGW as an application example of the aforementioned technology.

### Graphene doped RGW power divider

**Figure 5:**
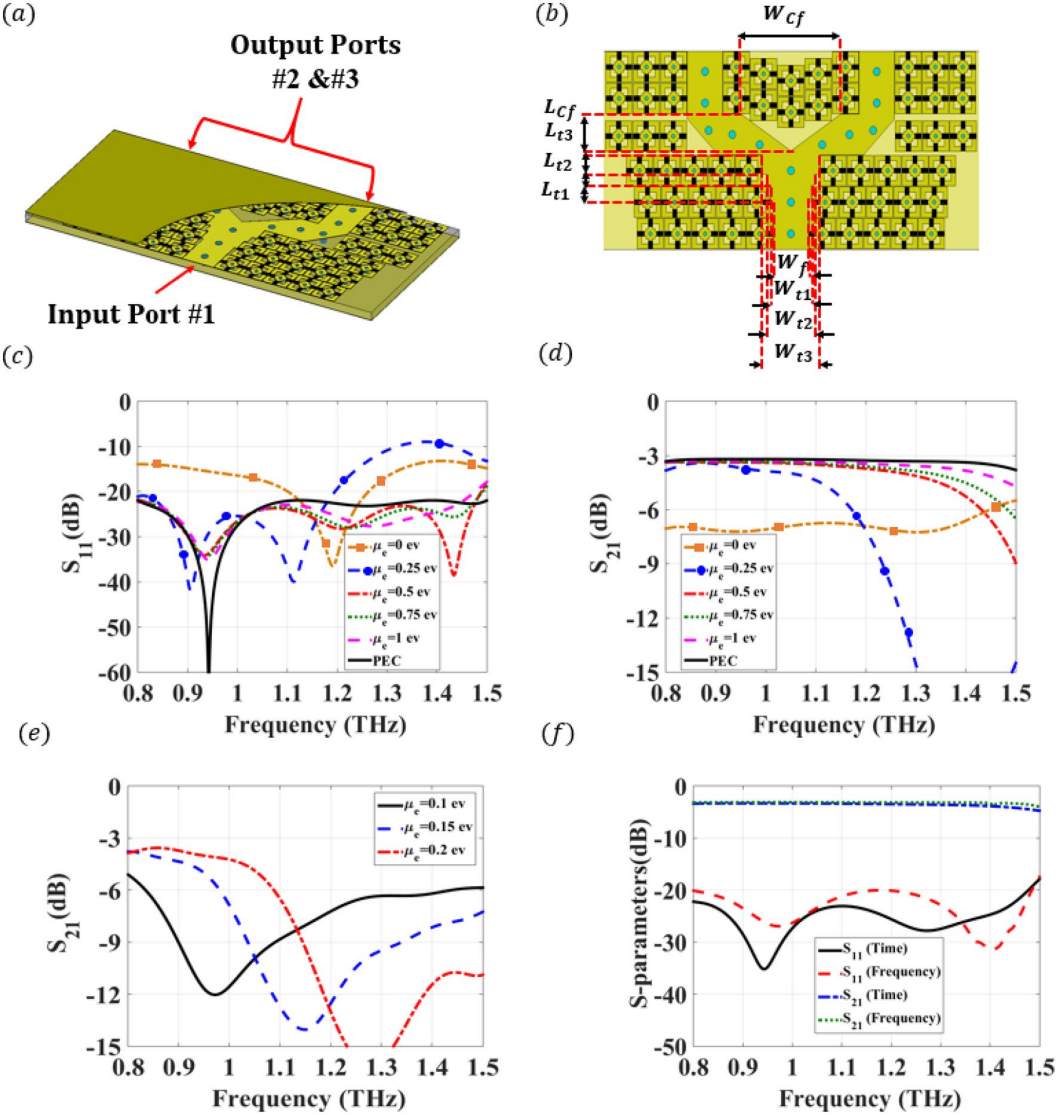
3-D configuration of the reconfigurable power divider and simulated S-parameter using full wave analysis. (**a**) 3-D view of the power divider constructed using a graphene-based PRGW line. (**b**) Schematic of the reconfigurable power divider with the design parameters. These parameters are chosen as $$W_f$$ = 40 μm, $$W_{t1}$$ = 46.5 μm, $$W_{t2}$$ = 53.5 μm, $$ W_{t3}$$ = 65 μm, $$ W_{Cf}$$ = 113 μm, $$L_{t1}$$ = 16 μm, $$L_{t2}$$ = 11 μm, $$ L_{t3}$$ = 18 μm, and $$ L_{Cf}$$ = 36 μm. (**c**) Simulated $$S_{11}$$ of the proposed reconfigurable graphene-based terahertz PRGW power divider for altering the chemical potentials from $$\mu _c=0$$ ev to $$\mu _c=1$$ ev. (**d**) Simulated $$S_{21}$$ of the proposed reconfigurable graphene-based terahertz PRGW power divider for altering the chemical potentials $$\mu _c=0{-}1$$ ev. (**e**) Simulated $$S_{21}$$ of the proposed reconfigurable graphene-based terahertz PRGW power divider for altering the chemical potentials $$\mu _c=0.1{-}0.2$$ ev. (**f**) S-parameters validation using time and frequency domain solvers at $$\mu _c= 1$$ ev.

The geometrical configuration of the proposed T-junction power divider implemented using the graphene-based terahertz PRGW line that has been discussed in the previous sections is shown in Fig. 5a. The proposed power divider is designed to achieve equal power divisions, where a quarter wavelength multi-section matching transformer is deployed to achieve a deep matching level over 0.8–1.5 THz operating bandwidth. Detailed design parameters of the deployed matching transformer are depicted in Fig. 5b. The reconfigurable mechanism of the power divider is studied by changing the chemical potential from $\mu _c=0$ ev to $\mu _c=1$ ev, where the simulated matching level and transmission are shown in Fig.5c,d, respectively. It can be depicted that at $\mu _c=0$ ev, a − 10 dB matching level with large transmission loss (< − 8 dB) can be achieved, where there is no bandgap supported by the unit cell and accordingly no operating bandwidth. By increasing the chemical potential, the power starts to be transmitted to the output port through only 30$\%$ (0.8–1 THz) of the operating bandwidth. Further increasing of the chemical potential is expanding the bandwidth until reaches 100$\%$ (0.8–1.5 THz) at a voltage of $\mu _c=1$ ev. Fig. 5e shows another simulation for the transmission with higher resolution in terms of the applied chemical potential, which shows that the proposed approach is suitable for continuously reconfigured waveguides. This study emphasizes controlling the operating bandwidth by changing the chemical potential of the graphene. The proposed contribution is validated through a comparison between two different commercial numerical packages in Fig. 5f, where an excellent agreement is achieved. The proposed structure can be fabricated using a certain fabrication process, where a large area of graphene produced by chemical vapor deposition (CVD) directly on microwave compatible with SiC substrates or Cu foils^21,46,47^.

## Conclusions

The investigation of the use of graphene in implementing a reconfigurable printed ridge gap waveguide (RPRGW) at terahertz has been proposed in this article. A printed ridge gap waveguide has well-known advantages compared with other guiding structures including low radiation and material loss, which is suitable for very high frequencies. An electronic model of graphene at terahertz has been studied in detail, where an infinitesimally thin surface characterized by surface conductivity has been deployed to model the graphene. A novel unit cell structure has been proposed, where some metallic parts have been replaced by graphene to achieve a reconfigurable electromagnetic band gap by changing the chemical potential. In addition, the graphene is not allocated in the signal path, which leads to a featured reconfigurable configuration with minimal losses. An innovative mathematical methodology based on the scattering parameters has been proposed to extract the dispersion relation and study the effect of the graphene material on the band gap of the unit cell. As an application example of the proposed technology, a dynamic PRGW line, and power divider has been proposed, where the effect of altering the chemical potential on operating bandwidth has been studied. For the future extension, this work can be applied to different guiding structures such as groove waveguides and substrate integrated waveguides, however, more losses will occur.

## Data Availability

The datasets used and/or analysed during the current study available from the corresponding author on reasonable request.
